# Influence of cellulose nanofiber fluid on flow instability and heat transfer of two-phase closed thermosyphon

**DOI:** 10.1016/j.heliyon.2023.e20925

**Published:** 2023-10-13

**Authors:** Chan hee Lee, Seong-Won Seo, Dong Kyou Park, Kwon-Yeong Lee

**Affiliations:** a Handong Global University, 558, Handong-ro, Heunghae-eup, Buk-gu, Pohang-si, Gyeongsangbuk-do, 37554, Republic of Korea; b Korea University of Technology and Education, 1600, Chungjeol-ro, Byeongcheon-myeon, Dongnam-gu, Cheonan-si, Chungcheongnam-do, 31253, Republic of Korea

**Keywords:** Two-phase closed thermosyphon, cellulose nanofiber fluid, geyser phenomenon, Flow instability, Heat transfer

## Abstract

In this research, the geyser phenomenon occurring in a small-diameter two-phase closed thermosyphon (TPCT) was observed and the instability of the device was discussed. Geyser phenomena interfere with the natural circulation of internal working fluids, increasing the thermal resistance of the system and contributing to the instability of the device. This study attempts to improve the thermal performance and stability of the system using cellulose nanofiber (CNF) fluid as the working fluid. The use of CNF fluid was observed to reduce the magnitude of temperature change inside the evaporator of the TPCT significantly. Moreover, it improved the local boiling heat transfer coefficient by 3.1 %, 87.3 %, and 181.2 % on average when the filling ratios are 0.25, 0.5, and 0.75, respectively. Studying the local heat transfer performance and instability will be helpful in designing a more stable TPCT efficiently. Additionally, the findings of this study can be applied to solar thermal power generation or heat pipe research for cooling strategies in computing servers, depending on the input heat load.

## 1 Introduction

A heat pipe (HP) is a device in which the working fluid is contained in a closed channel. It is a heat exchange equipment that transfers heat efficiently among devices through thermal conductivity and phase-change principles. The working fluid in an HP, which has a wick structure, liquidizes in a condensation section. Subsequently, the fluid moves back to the evaporation section through capillary force. Therefore, the HP can be operated without external power [1]. With technological development, the performance of machinery and electronic devices has improved. Moreover, with the development of device miniaturization, effective thermal management has become one of the most important tasks. Generally, HPs are used to remove heat from small products, such as central processing units. However, research on its use in various fields is also being actively conducted.

A two-phase closed thermosyphon (TPCT) is a wickless structure HP device. It requires the condensation section to be higher than the evaporation section because the condensate moves back to the evaporation section by gravity [2]. For thermal management, TPCTs are widely used in various fields owing to their simple structure, low thermal resistance, high efficiency, and low manufacturing cost. Accordingly, many studies have been conducted to investigate the TPCT limitations in heat transfer performance and how the device can be improved. Various filling ratios (FRs) of the working fluid were applied to TPCTs with various lengths of evaporation section. The best heat transfer performance corresponding to the FRs was observed to differ according to the length of the evaporation section [[3], [4], [5], [6]]. To improve the heat transfer efficiency of the system, studies on the use of other working fluids for the TCPT have been conducted [[7], [8], [9], [10]]. In particular, nanofluids with excellent thermal properties have been investigated [[11], [12], [13]]. In addition, the operating limit of the system (i.e., critical heat flux (CHF)) must be improved [14,15].

Nanofluids are solutions in which nanometer-sized particles are evenly suspended and dispersed in a base fluid. They exhibit excellent thermal conductivity and expansion characteristics [16]. However, inorganic nanoparticles have not been successfully used owing to the unstable dispersion caused by aggregation and sedimentation in long-term experiments [17,18]. Cellulose nanofiber (CNF) is an organic nanomaterial which is eco-friendly, lightweight, and nontoxic. Because CNFs have a negative charge when dispersed in water, they evenly spread in water, and particle dispersion is stable; hence, the potential occurrence of aggregation and sedimentation is considerably low [19]. In a previous study, the heat transfer performance of the TPCT is observed to improve when CNF is used as the working fluid; aggregation and sedimentation are not observed [20].

In the TPCT, rising steam (due to pressure difference) and falling condensate (due to gravity) meet. If steam is rapidly generated owing to the narrow inner diameter of the channel and high input power, an entrainment limit is reached [21]. In this case, the rising steam is unable to push down the condensate into the evaporation section. Moreover, the heat in the evaporation section is not efficiently transferred, and then a dry-out phenomenon occurs. Such an entrainment limit may be prevented by adjusting the FR and input power of the device. However, selecting an inner diameter that is less affected by the entrainment limit is crucial. Many studies have described the repeated occurrences and failures of these limits as confining effects [[22], [23], [24]] or the geyser boiling phenomenon [[25], [26], [27]]. Geyser phenomenon is an instability that occurs when there is not enough heat input to maintain a constant boiling in the evaporator [28,29]. Confinement number (CO) is a dimensionless variable for the diameter of a channel and has a close relationship with geyser phenomenon. Choi et al. [30] designed a TPCT device that satisfies Co = 0.245 in the range of 0.12<Co < 0.34 where geyser phenomenon occurs and used deionized (DI) water and CNF fluid as working fluid, and experiments were performed by applying super-hydrophilic surface modification to the evaporation section. In boiling of CNF fluid, small vapor bubbles are generated much faster than DI water. Through this experiment, it was confirmed that the total boiling heat transfer coefficient (BHTC) was improved in both CNF and super-hydrophilic surface modification. Previous studies on Geyser boiling phenomena occurring inside TPCT that affect its operating performance and design have been actively conducted so far. The boiling process inside TPCT consists of bubble nucleation, growth and detachment of bubble, and it was found through experiments that it is divided into low nucleation frequencies, transition regimes, and high nucleation frequencies, depending on the range of nucleation frequencies. In the low-frequency boiling regime, because the waiting and boiling phases were clearly distinguishable, the generation of large bubbles whose diameter was similar to that of the device was observed; uneven and unsteady characteristics were also noted. Next, at high nucleation frequencies, fully developed boiling characterized by fluid turbulence in the evaporator occurred because of the generation of small bubbles detaching from the heated surface. In the fully developed boiling section, extremely small fluctuations were observed [31]. Geyser boiling is a phenomenon that occurs at a low input power, high FR, and low pressure [[32], [33], [34]]. In the geyser boiling regime, periodic temperature fluctuations and large amplitudes were observed [30,33]. The regime proceeds in the following order: boiling delay, superheated boiling, explosive vapor eruption, and liquid refilling. The wall temperature of the evaporator abruptly increased during explosive eruption and then sharply dropped during liquid refilling [34,35]. In the previous study, Choi [20,30] evaluated the total performance of TPCT. However, in this paper, the characteristics of flow instability and the local heat transfer inside TPCT that change with FR were studied by analyzing each of the three thermocouples attached within the evaporation part of TPCT. In addition, it was compared and analyzed how much TPCT's stability and heat transfer capability improve compared to DI water when CNF fluid is used. Studying the local heat transfer and instability phenomenon will be helpful to design more stable TPCT efficiently. On the other hand, the previous studies about TPCT have mainly considered the total thermal resistance to consider the performance of TPCT. The results of this study are suitable for applications such as solar thermal power generation that utilize TPCT, and they can also be utilized in research on heat pipes for cooling strategies in computing servers [36], depending on the input heat load. In heat pipes like TPCT, entropy generation is also an important consideration. The entropy generation in a heat pipe can affect the overall thermal resistance of the heat pipe due to frictional losses and finite temperature difference in the flow of the working fluid. In effect, TPCT provides a larger temperature difference, which results in a larger amount of heat transfer and more entropy in the system. The entropy generation rate can be used to quantify the irreversibility of the system which is directly related to the lost work during any process [37]. However, since this study focuses on local thermal resistance and instability, entropy generation was not considered deeply in this study.

## 2 Experiment

### 2.1 Cellulose nanofiber fluid

The CNF used in this experiment was purchased from A & Poly; it was in gel form with a concentration of 2 wt%. The mass concentration of the CNF fluid used in this experiment was 0.5 wt%. In a wire pool boiling study [30] the CHF of the system improved by 56.5 % using CNF fluid (concentration: 0.5 wt%); this increase is higher than that when DI water was used. The measured properties of the 0.5-wt.% CNF fluid are listed in Table 1 [20]. The acidity of the CNF fluid (pH = 7.42) was neutral, and its electrical conductivity of 1.658 mS/cm considerably exceeded that of DI water (0.0005 mS/cm). Its density, 0.9984 g/cm^3^, does not considerably differ from that of DI water (0.9970 g/cm^2^). However, its viscosity (0.0089 N s/m^2^) was larger than that of DI water (0.00089 N s/m^2^) [20]. The following procedure was implemented to prepare a CNF solution with a concentration of 0.5 wt%. First, a lump of coagulated CNF with a 2-wt.% concentration was stirred until it became liquid. And, it was added after calculating the amount of water in consideration of the CNF mass so that the concentration was 0.5 w.t%. After mixing so that CNF can be spread in water, the process of dispersing the mixture for 30 min using an ultrasonic homogenizer (HUH-606, Hantech) was performed twice for a total of 60 min. As seen in the previous studies [30,39], sedimentation was not observed on CNF at 0.5 w.t% even after several months. Furthermore, during multiple boiling experiments using CNF at 0.5 w.t%, sedimentation, aggregation and segmentation didn't occur. These results confirm that CNF is sufficiently stable as a working fluid.

**Table 1:** Properties of CNF fluid and DI water [20].

Property	pH	Conductivity [mS/cm]	Density [g/cm^3^]	Viscosity [N-s/m^2^]	Fiber width [nm]	Zeta potential [mV]
CNF fluid (0.5 w.t.%)	7.42	1.658	0.9984	0.0089	1–20	−92.83
De-Ionized Water	7	0.0005	0.9970	0.00089	–	−30 ∼ −40

### 2.2 Experimental apparatus and procedure

**Fig. 1:**
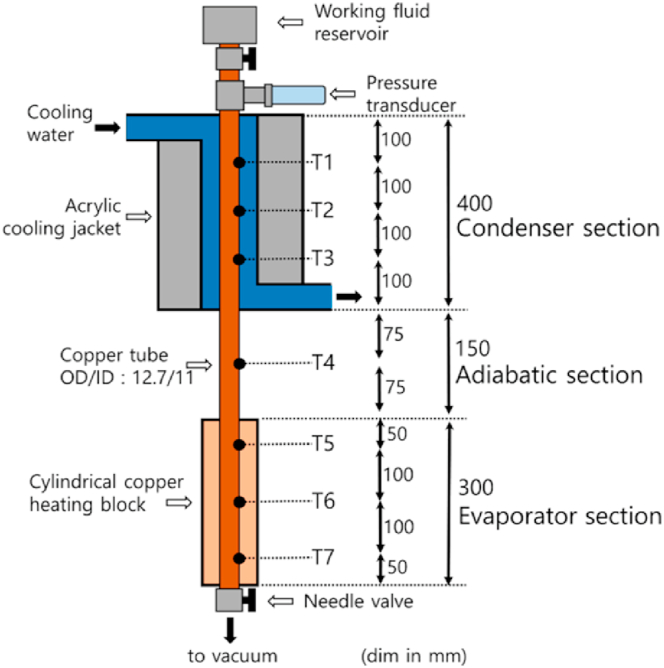
Experimental apparatus [20].

**Fig. 2:**
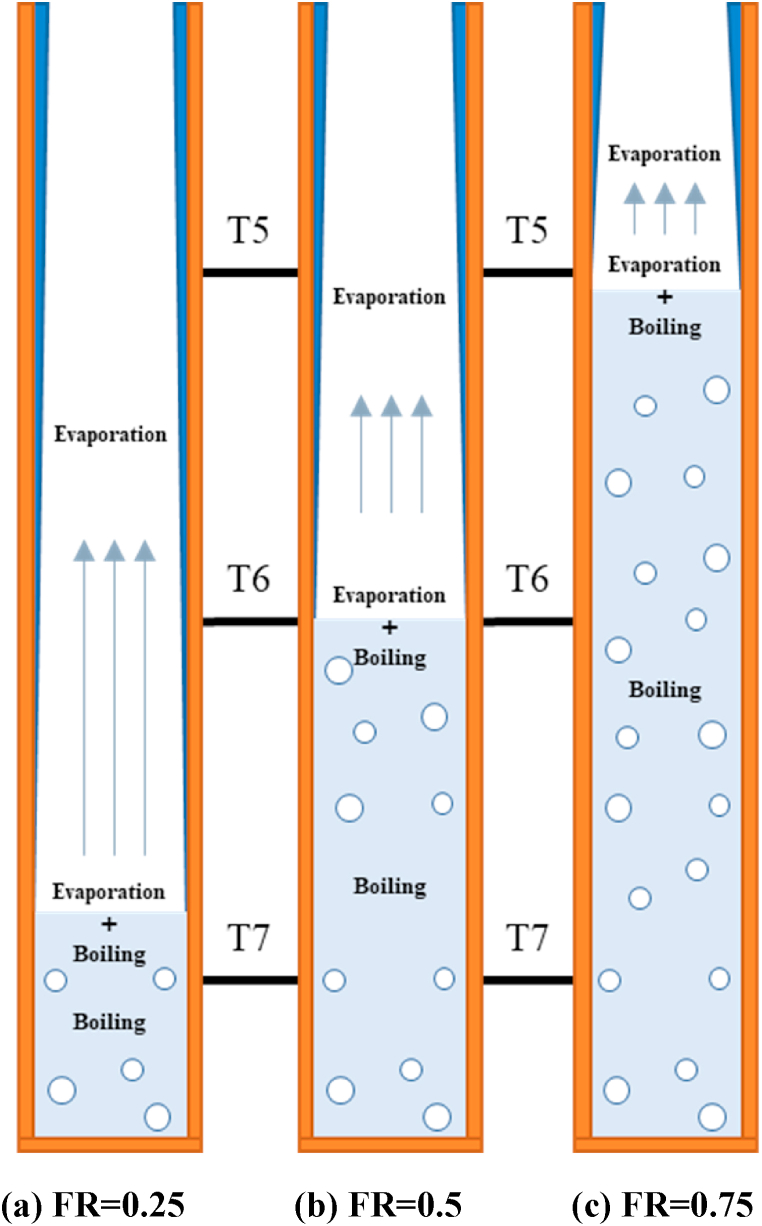
Schematic of flow regime in evaporator by filling ratios (FR = 0.25, 0.5, 0.75).

A schematic of the TPCT used in this experiment is shown in Fig. 1. The dimensions of the TPCT are as follows: the lengths of the evaporator, adiabatic component, and condenser are 300, 150, and 400 mm, respectively. The material of channel and the heater providing heat to the evaporator were copper. Owing to the structural characteristics of the evaporator channel and copper heater, the space between the two was filled with aluminum nitride powder (thermal conductivity: 150 W/mK) to minimize the contact thermal resistance. To supply heat to the evaporator, the heater was connected to a power source (N8953A, Keysight). The condenser was cooled by cooling water flowing through the cooling jacket. The acrylic cooling jacket was supplied with coolant at 20 °C and flow rate from a connected chiller (GR-C-00050A, Busung) was 0.5965 kg/s measured by flowmeter (Yuyu inst.). Three, one, and three thermocouples (Omega) were attached to the outer channel surfaces of the evaporator, adiabatic and condenser sections, respectively. Temperature data from the thermocouple were collected using a data acquisition module (34970A, Keysight) connected to the thermocouple. A pressure transmitter (PSHJ1000TCTJ, Sensys) was attached to the condenser to measure the internal pressure of the thermosyphon. The experimental procedure is as follows. The vacuum pump connected to the valve at the bottom of the evaporator part was operated with an internal vacuum of 0.02 bar; then, the valve was closed. Subsequently, the valve installed on the upper part of the device was opened and a certain amount of working fluid was injected by filling ratio (FR = 0.25, FR = 0.75). Fig. 2 shows the order in which the geyser effect occurs in the evaporator and the schematic of the boiling regime in the evaporator. Fig. 2(a) shows that evaporation and boiling occur near T7, and pure evaporation occurs near T5 and T6. Fig. 2(b) shows the flow regime inside the evaporator at FR = 0.5. Near T6, evaporation and boiling occur simultaneously; at T7, pure boiling; and at T5, pure evaporation. Next, Fig. 2(c) shows that evaporation and boiling occur near T5, and pure boiling occurs near T6 and T7. After injecting the working fluid, the heater (Woori heater) was powered by a supplier. The input power was increased from 100 to 800 W at 100-W increments. Input power was supplied for approximately 45 min to reach a steady state, and the temperature data were collected at 1-s intervals.

The temperature of the outer surface of the evaporator section was measured using thermocouples. The inner surface temperature is calculated using Equation (1).

(1) $$T_{e,i}=T_{e,o}-\frac{Q_{in}\cdot \log\left(\frac{D_{o}}{D_{i}}\right)}{2\pi L_{e}k_{copper}}$$

For the case of local BHTC, it was determined using the formula derived from previous studies [20,30] to calculate the total BHTC. The heating section was divided into three parts of equal length.

The local BHTC is calculated using Equations (2), (3):

(2) $$Q_{in}=V\cdot I$$

(3) $$h_{e,i}=\frac{Q_{in}}{A_{e,i}\left(T_{e,i}-T_{sat}\right)}$$

where $Q_{in}$ is calculated using the recorded voltage (V), current (I), and local temperature ($T_{e,i}$). The saturation temperature, $T_{sat}$, of the working fluid is based on the saturation pressure inside the channel and obtained using a pressure transducer.

During the steady state conditions, the temperature of the outer surface of the insulation was measured and found to be less than 1 °C to the room temperature. The instrument used to measure the external surface temperature of the insulation material is the same type as the thermocouples(K-type, Omega) used to measure the temperatures at T1∼T7. All the thermocouples have been calibrated using a temperature liquidbath calibrator (TP3M165E.2, Sika) and a calibrated RTD (Resistance Temperature Detector, Omega). As a result, the thermocouples have an uncertainty of ±0.15 °C.Therefore, insulation is sufficiently achieved to the extent that heat loss can be disregarded, and the heat loss was not considered in the data processing.

Table 2 shows the uncertainties of the data measurement devices, which is used in this study. The uncertainties of each experimental result obtained using the uncertainty calculation formula, Equation (4), is shown in Table 3 [38]. The uncertainty of the input heat was 0.28 %, and the uncertainty of the local BHTC was within 15 %. However, two points of BHTC data (100W with CNF on T5) with uncertainties exceeding 15 % were discarded.

(4) $$\delta f=\sqrt{\sum_{i=1}^{n}{\left(\frac{\partial f}{\partial x_{i}}\delta x_{i}\right)}^{2}}$$

**Table 2:** Uncertainties of measuring devices.

Measurement	Type of device	Uncertainty
Wall Temperature	Thermocouple (K-type, Omega)	±0.15°C
Pressure	Pressure transducer (PSHJ1000TCTJ, Sensys)	±0.15%
Voltage and Current	Power supply (N8953A, Keysight)	±0.2%
Coolant Temperature	Chiller (GR-C-00050A, Busung)	±0.15°C
Coolant Flow Rate	Flowmeter (Yuyu inst.)	±1%

**Table 3:** Uncertainties of experimental results.

Result	Symbol	Uncertainty
Input power [W]	$Q_{in}$	0.28 %
Local BHTC [kW/m^2^]	$h_{e}$	4.68–14.46 %

### 2.3 Frequency analysis

**Fig. 3:**
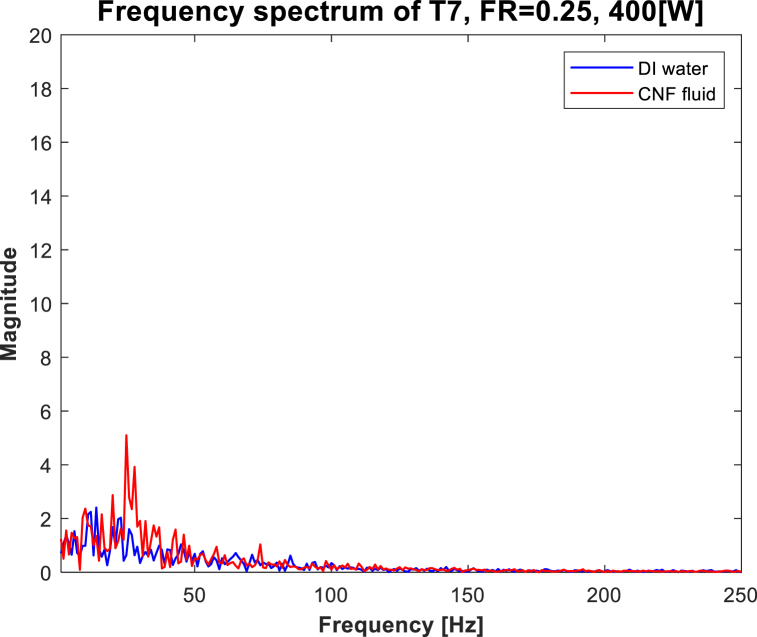
Frequency spectrum (FR = 0.25, T7, 400 W).

Frequency analysis of raw temperature data was performed to determine the pattern of the temperature data collected through the thermocouples attached to the evaporator. To convert the raw temperature data into signals with a finite number of discontinuities, the FFT (fast Fourier transform) algorithm of MATLAB was applied to the data. The FFT algorithm with a sampling frequency of 500 Hz was applied to 500 raw temperature datapoints reaching a steady state at every 100-W section. An example of the conversion process is shown in Fig. 3. In the figure, the frequency spectra corresponding to the use DI water and CNF fluid are plotted. First, to analyze the frequency characteristics of each case, the data in a band above a specific frequency (i.e., 50 Hz) were removed to derive a meaningful value. Then, five frequencies were set as dominant frequencies among the datapoints included in the section below 50 Hz according to the order of the largest frequency magnitude. Then, by setting the average frequency and magnitude of the five upper frequencies as the x (frequency band) and y (corresponding frequency signal) axes, the frequency characteristic for every 100-W section was represented.

## 3 Results and discussion

### 3.1 Wall temperature

**Fig. 4:**
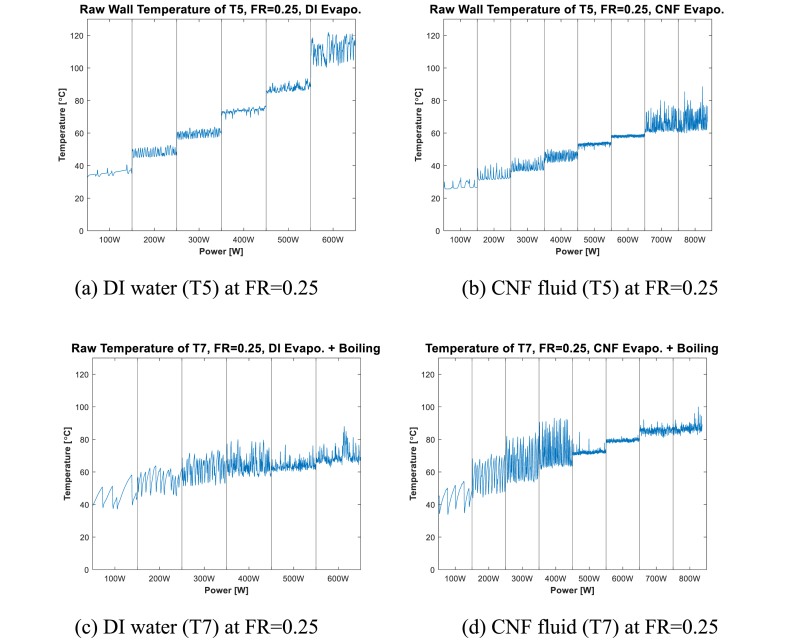
Evaporator wall temperature at FR = 0.25.

**Fig. 5:**
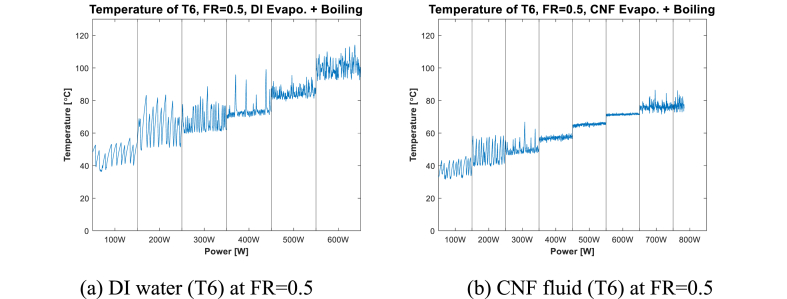
Evaporator wall temperature at FR = 0.5.

**Fig. 6:**
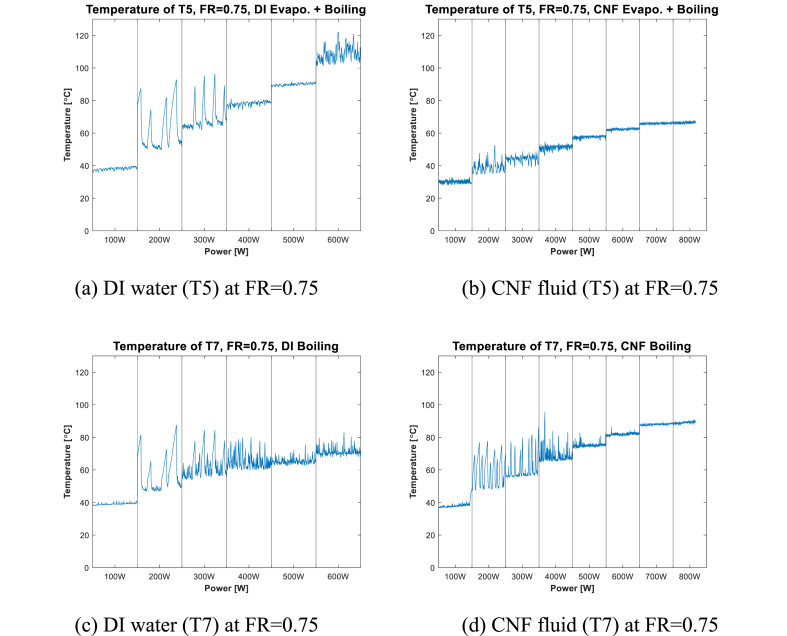
Evaporator wall temperature at FR = 0.75.

In a previous study, Choi et al. [20] reported that when the evaporator surface condition was bare and the input power was 200 W, a fast geyser phenomenon occurred regardless of the working fluid. Table 4 summarizes how the main phenomena of evaporation and boiling change according to FR. Fig. 4, Fig. 5, Fig. 6 show the local temperature of the internal surface of the channel in the evaporation section (at T5, T6, and T7) for each FR, depending on the working fluid (DI water and CNF fluid). The local temperature of the evaporation section showed a slightly different pattern depending on the location and input power. CNF generates small bubbles when boiling occurs and exhibits enhanced evaporation compared to DI water due to its water-containing ability [20].

**Table 4:** Phase-change heat transfer mode at local positions according to FR.

Temperature	FR		
	0.25	0.5	0.75
**T5**	Evaporation	–	Evaporation + Boiling
**T6**	–	Evaporation + Boiling	–
**T7**	Evaporation + Boiling	–	Boiling

First, Fig. 4 shows a comparison of the temperatures at T5 when FR = 0.25. Fig. 4, (a) and (b), indicates where pure evaporation occurs. The geyser effect with a long period was observed at 100 W for all cases, and the fast geyser effect, which had a short period, was observed in the 200–400-W section. Next, as shown in Fig. 4(b), the fluctuation in the 500–600-W section is low. Beyond 600 W, as shown in Fig. 4(a), “dry-out” occurs. In Fig. 4(b), a “near dry-out” phenomenon in which considerable temperature overshoot and oscillation occur is observed in the power section (from 700 to 800 W) [33].

Fig. 4 also shows the case of T7 when FR = 0.25; evaporation and boiling occur simultaneously. As shown in Fig. 4, (c) and (d), a geyser effect with a long period and large amplitude is observed in all cases at 100 W. A fast geyser effect with a large amplitude is observed in the 200–400-W section, as shown in Fig. 4, (c) and (d). At this section, the use of CNF fluid results in a geyser effect with a larger amplitude than that in the other cases in which DI water was used as the working fluid. Beyond the 400-W section, a churn boiling regime in which small and irregular oscillations occur is observed. As indicated by the graph in Fig. 4(d), in the 700–800-W section, more irregular oscillations are than those in the previous power section. This is because of the water-containing ability of CNF, as explained earlier, which promotes more vigorous evaporation [20].

Fig. 5 shows the temperature (T6) of the evaporator wall (where boiling and evaporation occur) at FR = 0.5. As shown in Fig. 5(a), the geyser effect, which has an irregular period and large amplitude, is observed in the low-power section (200–300 W). Beyond 300 W, the amplitude exhibited a decreasing pattern; however, after 600 W, the temperature measurement stopped owing to the rapid temperature increase. In contrast, in the case shown in Fig. 5(b) in which CNF fluid is used, the amplitude of the geyser effect is smaller than that when DI water is used in the low-power section. Subsequently, the temperature change stabilized.

Next, Fig. 6, (a) and (b), shows the case at T5 (FR = 0.75) in which evaporation and boiling occur simultaneously. The geyser effect in Fig. 6(a) has a larger period and amplitude than that shown in Fig. 6(b) at the 200–300-W section. Beyond 300 W, the temperature graph in Fig. 6(b) shows a churn boiling regime until “dry-out” occurs. With the use of DI water, a pattern similar to that shown in Fig. 6(a) at the 400–500-W section is observed. Beyond the 600-W section, the CHF values are 600 and 800 W, as shown in Fig. 6, (a) and (b), respectively.

In Fig. 6(c) and (d), where pool boiling occurs, the geyser effect is observed to occur in all cases in the 200–400-W section. In the 200-W section (Fig. 6(c)), the use of DI water results in a geyser effect whose amplitude is longer than that shown in Fig. 6(d). This confirms that CNF suppresses the geyser phenomenon due to its ability to generate smaller bubbles compared to DI water when boiling occurs [20]. Beyond the 400-W section, when CNF fluid is used, churn boiling is observed (Fig. 6(d)); this is similar to the case shown in Fig. 6(b).

The stability resulting from the use of CNF fluid and DI water can be compared by analyzing the temperature graph at the local point where evaporation, simultaneous evaporation and boiling, and boiling occur (Fig. 4, Fig. 5, Fig. 6). As shown in Fig. 4, (a) and (b), the resulting difference in stability between using CNF fluid and DI water as working fluid is not distinct. As shown in Fig. 6, at the point where boiling occurs, the temperature graph confirms that the period of temperature change at the low-power section (200–300 W) using CNF fluid is shorter than when DI water is used. This is because at low power, more small bubbles are formed and broken by CNF fluid than by DI water; consequently, more active pool boiling occurs [39]. Next, in Fig. 4 ((c) and (d)), 5 ((a) and (b)), and 6 ((a) and (b)), which show the temperature graph of the part where evaporation and boiling occur simultaneously, the stability of temperature change is found to improve when CNF fluid is used in the low-power section (200–300 W).

### 3.2 Frequency spectrum

**Fig. 7:**
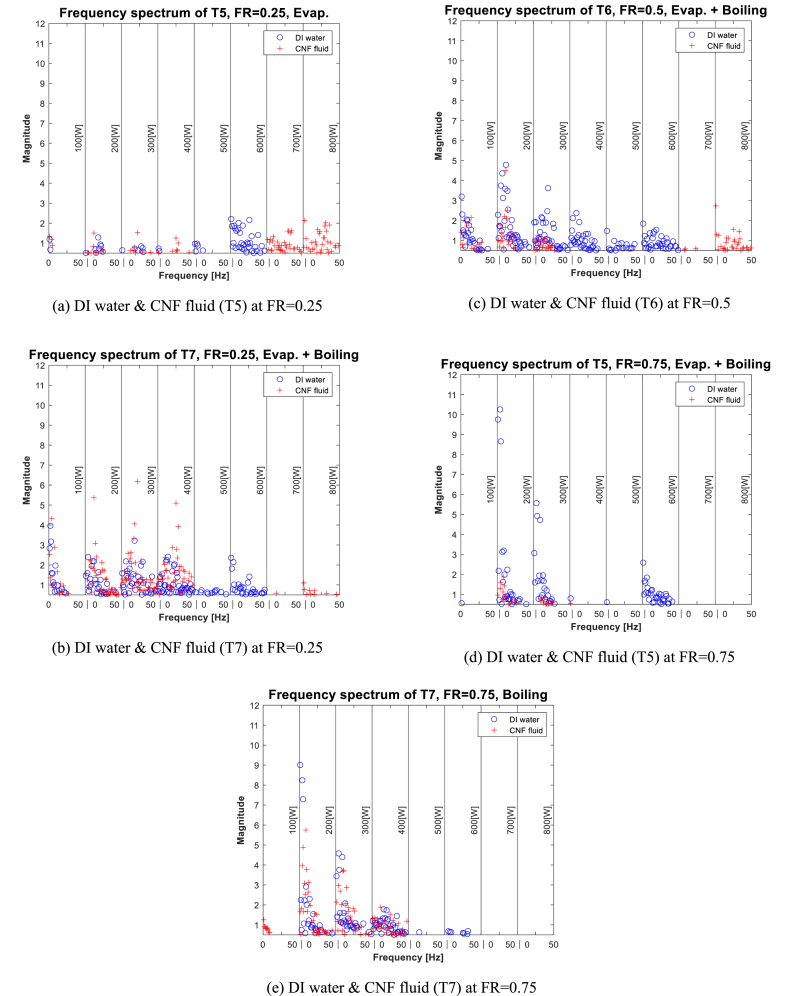
Frequency spectrum of Evaporator at 100[W]-800[W].

Fig. 7(a)–(e), shows the frequency spectrum of the evaporator wall temperature when FR = 0.25, 0.5, and 0.75. First, as shown in Fig. 7(a), when evaporation occurs at FR = 0.25, the overall graph pattern is similar regardless of the type of working fluid in the 100–400-W section. Beyond 500 W, the “dry-out” phenomenon occurs inside the TPCT. Heat exchange is not normal; consequently, instability in temperature change is observed.

Next, in Fig. 7(b), evaporation and boiling occur simultaneously. The power magnitude when CNF fluid is utilized is similar to that when DI water is used in the 100–400-W section. However, when CNF fluid is used, some of the frequencies are large compared with the maximum frequencies when DI water is used. In contrast, as shown in Fig. 7(c) and (d), as FR increases, the improvement in stability is better when CNF fluid is used that when DI water is utilized at the 200–300-W section. In Fig. 7(e), when pool boiling occurs, all frequencies are large at the 200–300-W section.

The results of analyzing the surface temperature data of the evaporator and frequency data are as follows. First, at the internal point of the evaporator where evaporation or boiling occurs, the frequency component is similar at the 200–400-W section regardless of whether CNF fluid or DI water is used. In addition, at the 400–800-W section, the use of CNF fluid was observed to improve stability as the frequencies decreased at the point where the boiling phenomenon occurred. Next, when the CNF fluid is used, the stability at the point where evaporation and boiling occur simultaneously in the TPCT improves, as confirmed by frequency analysis. In the 200–400-W section, the frequency magnitudes when the CNF fluid is utilized decrease significantly compared with those when DI water is used (except when FR = 0.25). Beyond the 400-W section, low temperature magnitude was maintained regardless of the FR, as confirmed by the graph.

### 3.3 Heat transfer

Choi et al. [20] compared the BHTC of the total evaporation section when FR = 0.25, 0.5, and 0.75 and showed that the BHTC of the TPCT when CNF fluid was used improved.

**Fig. 8:**
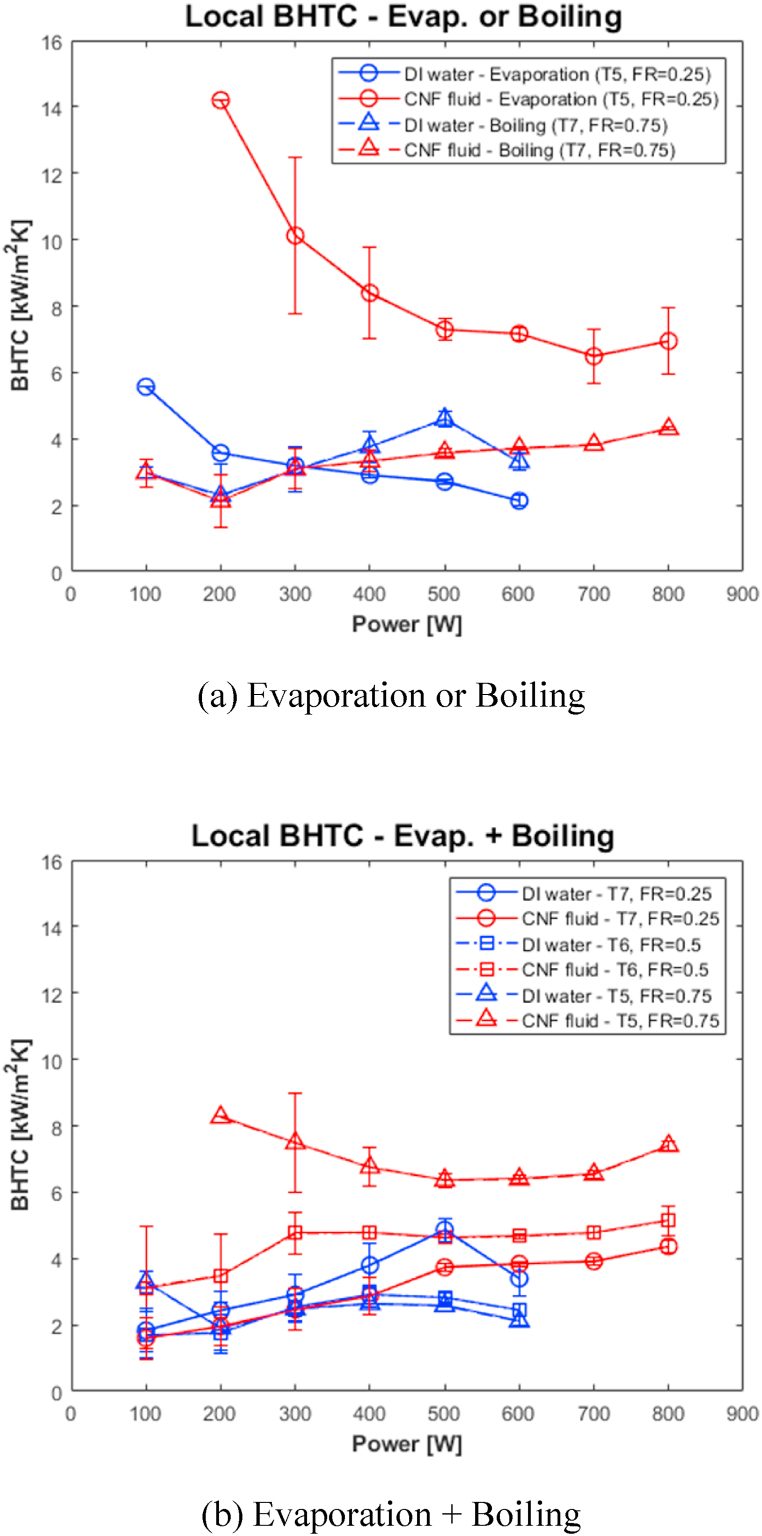
Local BHTC of Evaporator at all FR conditions.

Fig. 8, (a) and (b), compares the local BHTCs at T5, T6, and T7 when FR = 0.25, 0.5, and 0.75. In Fig. 8(a), in the case of the 100–200-W data, the temperature deviation between T_sat_ and the wall temperature at T5 is large owing to the geyser phenomenon. Therefore, because a case in which the standard deviation is excessively large exists, the error bar of the large standard deviation case of the 100–200-W data is omitted in this study. Moreover, in Fig. 8(b), the local BHTC at T5 using the CNF fluid is not plotted in the 100-W section. This is because the internal surface temperature, $T_{e,5}$, at T5 is measured to be lower than $T_{sat}$ such that the BHTC is negative or $T_{e,5}$ slightly exceeds $T_{sat}$; consequently, the measured BHTC is excessively high. At 100 W, the temperature at T5 was low because the cooling effect of the condensate exceeded the heating effect of the heater, and the heat flux ($q^{\prime \prime }$) was not large. In this case, $T_{sat}$ is 27.1 °C, and at this point, the condensate temperature will be between the cooling water temperature of 20 °C and the adiabatic section temperature of 24.35 °C. Therefore, when the condensate flows down to the T5 thermocouple, there may be cases where the subcooled condensate has a temperature lower than T5.

As shown in Fig. 8(a), the local BHTC improves on average by 158.9 % and 1.0 % at T5 and T7 when FR = 0.25 and 0.75. As shown in the graph, at T5, when the CNF fluid is used and FR = 0.25, the BHTC improves owing to the characteristics of the CNF fluid whose capacity to hold water is considerable. Therefore, if the CNF fluid adheres to the evaporation surface, more heat can be transferred. As shown in Fig. 8(b), the local BHTC improved by 3.1 %, 87.3 %, and 181.2 % on average when FR = 0.25, 0.5, and 0.75, respectively. When DI water was used and as FR increased, the BHTC decreased; in contrast, when CNF fluid was used, the BHTC increased.

**Fig. 9:**
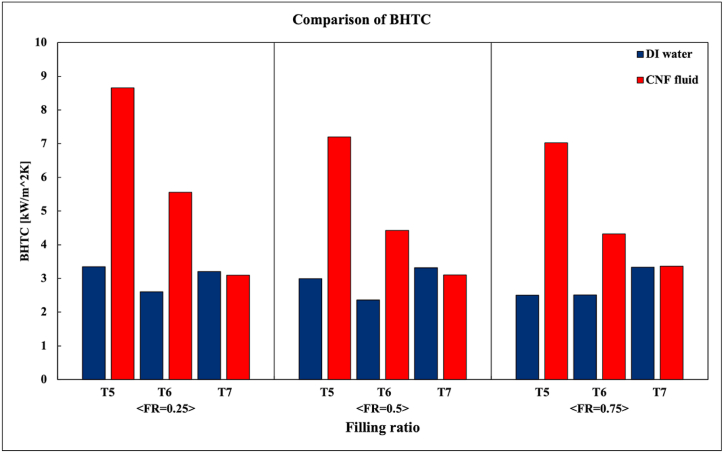
Comparison of Local BHTC at all FR conditions.

Fig. 9 compares and shows the local BHTC when FR = 0.25, 0.5, and 0.75. As shown in the graph, when FR = 0.25, the BHTC improves when CNF fluid is used in the T5 area where evaporation occurs. When FR = 0.75, the use of the CNF fluid does not significantly affect the T7 area where boiling occurs. However, the use of CNF fluid improved the BHTC as the FR increased when boiling and evaporation occurred simultaneously at T7 (FR = 0.25), T6 (FR = 0.5), and T5 (FR = 0.75).

## 4 Conclusions

Choi et al. [20] showed that the increase in the total BHTC was higher using CNF fluid than utilizing DI water as the working fluid of the TPCT. In this study, the effects of using CNF fluid were analyzed and compared with those when DI water was used in terms of the flow instability and local heat transfer performance of the TPCT system with Co = 0.245. Depending on the FR, the point at which evaporation and boiling occurred inside the evaporator of the TPCT changed. Accordingly, the effect of using CNF fluid was analyzed in terms of FR by dividing the evaporator into three parts (i.e., at T5, T6, and T7).

The results of this study are as follows.

(1) The use of CNF fluid compared with utilizing DI water reduces the temperature change of the graph as the FR and the input heat increases.
(2) The CNF fluid improves stability as the magnitude of the frequencies decreases at the point where the boiling phenomenon occurs. In the area where evaporation and boiling occur, the magnitude of the frequencies significantly decreases compared with that when DI water is used (except when FR = 0.25).
(3) The BHTC improved by 158.9 % at T5 on average when FR = 0.25. In addition, at the point where evaporation and boiling occurred simultaneously, the BHTC improved by 3.1 %, 87.3 %, and 181.2 % on average when FR = 0.25, 0.5, and 0.75, respectively.

In terms of instability, as a result of separately analyzing T5 and T7, where evaporation and boiling occur differently depending on the FR, the CNF fluid has the characteristic of reducing the amplitude of the geyser effect. Moreover, in terms of heat transfer, the use of CNF fluid is.

## Data Availability

Data will be made available on request.

## CRediT authorship contribution statement

**Chan hee Lee:** Writing – original draft, Visualization, Methodology, Investigation, Formal analysis, Data curation. **Seong-Won Seo:** Writing – review & editing, Writing – original draft, Validation, Software, Project administration, Methodology, Investigation, Formal analysis, Data curation. **Dong Kyou Park:** Writing – review & editing, Validation, Resources, Methodology, Investigation. **Kwon-Yeong Lee:** Writing – review & editing, Supervision, Resources, Project administration, Methodology, Investigation, Funding acquisition, Conceptualization.

## Declaration of competing interest

The authors declare that they have no known competing financial interests or personal relationships that could have appeared to influence the work reported in this paper.

## Abbreviations

A: surface area $\left[m^{2}\right]$
BHTC: Boiling heat transfer coefficient
$C_{p}$: specific heat [J/kgK]
$C_{\text{sf}}$: coefficient of surface (copper-water: polished)
CHF: Critical heat flux
CNF: Cellulose nanofiber
Co: confinement number
D: diameter [m]
DI: De-ionized
FR: Filling ratio
h: heat transfer coefficient $\left[W/m^{2}K\right]$
$h_{\text{fg}}$: enthalpy (gas-fluid) [J/kg]
I: current [A]
k: thermal conductivity coefficient [W/mK]
L: length [m]
p: pressure transducer
Q: heat transfer rate [W]
Re: Reynolds number
T: temperature [°C]
TPCT: Two-phase closed thermosyphon
V: voltage [V]

**Greek symbols**
α: temperature coefficient of resistance $\left[m^{2}\right]$
μ: viscosity $[\text{Ns}/m^{2}$]

**Subscript**
*cop*per: copper
e: evaporation
i: Inner surface
in: input
l: liquid
o: Outer surface
sat: Saturation
v: vapor
